# The Effect of Fracture Patterns, Pinning Configuration, Surgeon Experience and Subspecialty on Short-Term Radiological Outcomes of Pediatric Supracondylar Humeral Fractures Treated in the Prone Position: A Case-Series

**DOI:** 10.3390/healthcare11192648

**Published:** 2023-09-28

**Authors:** Andrea Vescio, Giovanni Carlisi, Vincenzo Roberto Macrì, Francesco Sanzo, Giuseppe Gigliotti, Daria Anna Riccelli, Giuseppe Tedesco, Michele Mercurio, Olimpio Galasso, Giorgio Gasparini, Garrett R. Jackson, Jorge Chahla, Filippo Familiari

**Affiliations:** 1 Departiment of Orthopaedic and Trauma Surgery, Azienda Ospedaliera Pugliese Ciaccio, 88100 Catanzaro, Italy; andreavescio88@gmail.com (A.V.); vincenzormacri@gmail.com (V.R.M.); francescosanzo@alice.it (F.S.); giuseppe.gigliotti59@gmail.com (G.G.); palanchina85@hotmail.it (D.A.R.); 04tedesco@gmail.com (G.T.); 2 Departiment of Orthopaedic and Trauma Surgery, Magna Graecia University, 88110 Catanzaro, Italy; dr.giovannicarlisi@gmail.com (G.C.); galasso@unicz.it (O.G.); gasparini@unicz.it (G.G.); filippofamiliari@unicz.it (F.F.); 3 Research Center on Musculoskeletal Health, MusculoSkeletalHealth@UMG, Magna Graecia University, 88100 Catanzaro, Italy; 4 Department of Orthopaedic Surgery, Rush University, Chicago, IL 60612, USA; garrett.jackson@rushortho.com (G.R.J.); jorge.chahla@rushortho.com (J.C.)

**Keywords:** children, elbow, supine position, prone position, radiological outcome, subspecialty, pediatric orthopedic, general orthopedic, pinning, CRPP, radiological exposure

## Abstract

Background: The most common treatment modality for supracondylar humerus fractures (SCHFs) in children is closed reduction and percutaneous pinning (CRPP). Nonetheless, debate persists regarding the optimal technique used. Therefore, the purpose of our study was to investigate the impact of surgeon experience, surgeon subspecialty and pin configuration on short-term radiological outcomes following CRPP of displaced SCHFs. Methods: Patients less than 14 years of age who underwent CRPP for displaced SCHFs in the prone position between January 2018 and December 2022 were analyzed. Patients were separated into subgroups based on fracture type (low vs. high sagittal), pin configuration (lateral, cross, other), number and configuration of K-wires and first operator surgical experience. The following outcome measurements were collected: postoperative Baumann angle (BA), Shaft-Condylar angle (SCA), surgical duration (SD), duration of radiation exposure (DRE) and number of clinical and radiological follow-ups (FU). Results: A total of 44 patients with a mean age of 6 ± 2.5 years were included in the final analysis. The mean post-operative BA and SCA were 74.8° ± 4.9° and 37.7° ± 10.2°, respectively. No significant differences were found in the post-operative Baumann’s angle or SCA among the subgroups. Regarding secondary outcomes, no differences were found among each subgroup regarding SD, DRE and FUs. Conclusion: Short-term radiological outcomes following the treatment of SCHFs treated in the prone position are not affected by fracture patterns and pinning configuration, regardless of the surgeon’s years of experience or subspecialty.

## 1. Introduction

Supracondylar humerus fractures (SCHFs) account for 50% to 70% of elbow fractures in children, typically occur between the ages of 3 and 10 years [1,2] and represent the first pediatric elbow injury [3]. These fractures are predominantly caused by extension trauma [4] and more common among males [5].

SCHFs in children requiring reduction and surgical fixation can be difficult to treat due to the potential for residual deformity. As per the Gartland classification, fractures greater than grade II necessitate surgical reduction [6]. However, there remains no consensus among orthopedic surgeons concerning the optimal intraoperative position of the patient, namely supine or prone [7]. The supine position has been the most commonly adopted approach during surgery [8]. Treatment for SCHF fractures in the supine position involves two steps: first, applying a reduction force at the level of the olecranon to bring the distal fragment back into alignment with the proximal fragment; and second, hyperflexion of the elbow to maintain reduction during fixation [8,9,10]. The elbow is hyperflexed, while the fracture is fixed [8,9,10]. Nevertheless, recent evidence has shown that the prone position is an efficient alternative [9]. The prone position leverages gravity to facilitate reduction and osteosynthesis, allowing the distal humerus to naturally align with the correct position in the sagittal plane. Notably, the avoidance of tourniquet application in this position circumvents potential hindrances during reduction maneuvers on the proximal fragment. Despite its advantages, the prone position has orthopedic and anesthesiologic disadvantages. Airway control may prove to be more challenging in this position, and if vascular access becomes necessary, the patient must be repositioned [10]. Based on the 2022 survey conducted by the European Pediatric Orthopedic Society (EPOS), 71.2% of pediatric orthopedic surgeons regard surgeon experience as being of “crucial importance” in determining expected treatment outcomes for supracondylar humeral fractures [11]. These findings underscore the significance of a surgeon’s experience, which directly influences outcomes, considering the notable learning curve associated with this procedure. In contrast, a recent study found no differences in adverse events at 30 days, reoperations or readmissions when comparing the performance of young and senior surgeons [12].

The most common treatment modality for SCHFs in children is closed reduction and percutaneous pinning (CRPP). Nonetheless, debate persists regarding the optimal technique used, i.e., crossed or lateral pinning, as well as the number of pins used. According to the 2022 EPOS survey, 84.8% and 75% of respondents consider pin configuration and the number of pins as “average” or “crucial” factors, respectively. However, the survey did not reveal a clear preference for pin configuration, with crossed pins (33.7%) and two divergent lateral pins (23.9%) being the most commonly used approaches. This observation highlights the existing uncertainty in the literature, which has not yet established a definitive gold standard, given the varying biomechanical [13,14,15] and clinical [16,17,18] studies reporting superiority of one-pin configuration over the others [19].

Therefore, the purpose of our study was to investigate the impact of surgeon experience, surgeon subspecialty and pin configuration on short-term radiological outcomes following CRPP of displaced SCHFs. The authors hypothesized that fracture pattern, surgeon experience and subspecialty would significantly influence short-term radiologic outcomes following SCHF treatment. Conversely, the authors hypothesized that pin configuration would not have a substantial impact on fracture reduction.

## 2. Materials and Methods

### 2.1. Study Design

According to the Consort Checklist, a retrospective review was performed using the medical records of children younger than 14 years of age who underwent CRPP for displaced SCHFs between January 2018 and December 2022 at Azienda Ospedaliera “Pugliese-Ciaccio”, Catanzaro, Italy. Patients meeting the following inclusion criteria were included in the study analysis: (1) confirmed diagnosis of closed Gartland type II or III SCHF; (2) patients < 14 years of age; (3) use of intraoperative prone position of the patient; (4) completed radiographic follow-up data. The exclusion criteria were as follows: (1) polytrauma patients with concomitant fractures; (2) patients > 15 years of age; (3) open or pathological fractures; (4) no complete radiographic data. 

### 2.2. Patient Evaluation and Data Analysis

Patient charts were retrospectively reviewed by two independent authors (**Initials blinded for peer review**). Patient demographics were collected using electronic medical records and included sex, age at the time of trauma, side involved, presence or absence of associated neurovascular injury, whether the fracture was open and the Gartland and Bahk classification [16,20,21]. The patient’s intraoperative position (prone, supine), pin configuration (lateral, cross, other), number and configuration of K-wires, duration of surgery (minutes), duration of x-ray exposure (seconds), first operator surgical experience (<10 or ≥10 years), first operator SCHF surgical treatment experience (15< or ≥15 CRPPS for SCHFs performed) and number of clinical and radiological follow-up visits were also obtained from medical records (Table 1).

#### Sub-Groups

The cohort was divided into the following subgroups and compared: (1) low sagittal fracture (based on the Bahk classification in lateral view), (2) high sagittal fracture (based on the Bahk classification in lateral view), (3) lateral pin configuration, (4) cross pin configuration, (5) other pin configuration, (6) “novice” first operator (defined by <10 years of surgical experience or <15 CRPPs for SCHFs performed, (7) “experienced” first operator (defined by ≥10 years of surgical experience or ≥15 CRPPs for SCHFs performed, (10) pediatric orthopedic surgeon (first operator) and (11) general orthopedic surgeon (first operator) (Table 1). 

### 2.3. Interventions

A closed reduction and percutaneous pin fixation were performed to treat all participants within 48 h following admission to the ER. The arm had been positioned at a “C” on the brightness amplifier plate after general anesthesia had been induced. A prone position was used during surgery, and 1.6 to 2 mm K-wire was employed for the fixation after the closed reduction. It was carried out using lateral or cross-pin configuration. When the cross-pin configuration was adopted, the initial pin was inserted in the exact center of the lateral condyle, and the subsequent wire was introduced medially while being careful to spare the ulnar nerve. A marker for the insertion of the medial wire was the little portion of the medial epicondyle where the pin must be placed as far anterior as possible to prevent iatrogenic nerve injury. When the lateral-pin configuration was chosen, the initial wire was positioned approximately parallel to the olecranon bone; the subsequent wire was introduced farther to the side. An adjunctive pin was inserted medially (two lateral and one medial pins) or laterally (three lateral pins) when persistent fracture instability was evident. The individuals were immobilized through an easy-to-use posterior splint at 90 degrees of flexion after being stabilized with pins. Optimizing perfusion and reducing edema were the objectives. K-wires protruded from the wound in each case. Generally, the treated subjects received their discharge the next day and then returned for a clinical and radiological examination 5 to 10 days later. The pins were typically removed four weeks following the surgery and after a radiological assessment, and every individual underwent a rigorous rehabilitation program to recover full elbow range of motion. Every patient included in the study was evaluated at one, three, six and twelve months after the surgery and each year until skeletal maturity.

### 2.4. Outcome Assessments

The primary outcomes of our study were the measured Baumann’s angle in the PA view and shaft-condylar angle in the lateral view. The Baumann’s angle is formed by the humeral axis and a straight line through the epiphyseal plate of the capitulum (normal value: 64–81 degrees; mean: 72 degrees; 75–80 degrees [20]). The shaft-condylar angle in the lateral view is defined as the angle between the axis of the humeral and the capitellum (normal value: <40° [21]). Measurements were performed on post-operative X-rays by two authors (GC and AV). The secondary outcomes included surgery duration in minutes, radiation exposure in seconds, complications and number of clinical and radiological follow-ups.

### 2.5. Statistical Analysis

In the case of continuous data, means and standard deviations were reported. Counts were utilized to represent categorical variables. The age means of the subgroups were compared using the ANOVA test. The homogeneity of the cohorts based on gender and side was confirmed using the χ^2^-test. *p* = 0.05 was used as the statistical significance standard. The 2016 GraphPad Software (GraphPad Inc., San Diego, CA, USA) was used for all statistical analyses.

## 3. Results

### 3.1. Sample and Subgroups

Forty-four patients (21 males and 23 females) out of fifty-six were eligible and enrolled in the study. A total of twelve patients were excluded, ten patients due to incomplete follow-up and two patients due to polytrauma. The mean patient age was 6.0 ± 2.5 years, and mean follow-up was 2.7 ± 1.9 years. Table 1 reports the number of patients within each subgroup.

According to the Bahk classification, in lateral view cohort subgroups, 28 patients were included in the low sagittal fracture (mean patient age was 6.2 ± 2.5 years and mean follow-up was 2.4 ± 1.5 years) and 16 subjects were included in the high sagittal fracture (mean patient age was 5.8 ± 2.6 years and mean follow-up was 3.2 ± 2.4 years). 

Thirteen patients were allocated in the lateral pin configuration cohort (mean patient age was 5.3 ± 3.1 years and mean follow-up was 3.1 ± 1.3 years), twenty patients were allocated in the cross-pin configuration group (mean patient age was 6.3 ± 2.0 years and mean follow-up was 2.5 ± 1.7 years) and eleven patients in the other pin configuration group (mean patient age was 6.4 ± 2.7 years and mean follow-up was 2.8 ± 2.9 years). 

According to the first operator experience, the 44 patients were divided in two groups: experienced first operator (28 patients; mean patient age was 6.1 ± 2.5 years and mean follow-up was 2.6 ± 1.4 years) and novice first operator (16 subjects; mean patient age was 6.0 ± 2.5 years and mean follow-up was 2.9 ± 2.6 years).

The patient age mean of 5.8 ± 2.3 years and 6.1 ± 26 years were recorded for the pediatric orthopedic surgeon and general orthopedic surgeon groups, respectively. The mean follow-up for the same subgroups were 2.8 ± 2.0 years and 2.4 ± 1.7 years, respectively.

No significant differences were found between the subgroups based on patient demographics (*p* > 0.05). Extended patient demographics comparison data are reported in Table 1.

### 3.2. Primary Outcome

Table 2 reports the postoperative radiological outcomes for all included patients. The post-operative Baumann’s angle for the entire cohort was 74.8 ± 4.9, and the post-operative SCA in lateral view was 37.7 ± 10.2. 

The post-operative Baumann’s angle for the low sagittal fracture group was 75.9 ± 8.9, and the post-operative SCA in lateral view was 37.8 ± 9.7; while the post-operative Baumann’s angle for the high sagittal fracture group was 71.6 ± 5.6, and the post-operative SCA in lateral view was 38.3 ± 11.1 (*p* > 0.05).

No significant statistical differences (*p* > 0.05) were found in the post-operative Baumann’s angle or post-operative SCA between lateral pin cohort (post-operative Baumann’s angle was 73.6 ± 5.7; and post-operative SCA mean was 40.0 ± 7.1), cross pin configuration group (post-operative Baumann’s angle was 75.9 ± 4.6; and post-operative SCA mean was 39.4 ± 11.2), and other pin configurations group (post-operative Baumann’s angle was 74.1 ± 6.6; and post-operative SCA mean was 32.2 ± 8.3). 

A mean of 75.1 ± 6.1 was recorded for the experienced first operator group for the post-operative Baumann’s angle and a mean of 74.3 ± 4.2 for the novice first operator group (*p* > 0.05). A mean of 39.2 ± 9.4 was noted for the experienced first operator group for the post-operative SCA and a mean of 35.0 ± 10.4 for the novice first operator group (*p* > 0.05).

According to the surgeon subspecialty, the post-operative Baumann’s angle means were 74.9 ± 7.1 and 74.6 ± 4.8 for the pediatric and general orthopedic surgeon groups (*p* > 0.05), respectively; the post-operative SCA means for the same group were 38.1 ± 11.1 and 36.4 ± 9.1 (*p* > 0.05). 

### 3.3. Secondary Outcome

No statistical differences were found among each subgroup regarding surgery duration, during of radiation exposure and number of clinical and radiological follow-ups (Table 3). Two patients were treated by required additional surgery for unsatisfactory reduction (Table 1).

## 4. Discussion

When treated in the prone position, short-term radiological outcomes following the treatment of SCHFs are not affected by fracture patterns and pinning configuration, regardless of the surgeon’s years of experience or subspecialty. No differences were found in operative time, radiographic exposure and number of clinical and radiographic follow-ups.

Based on the authors’ best knowledge, this article is the first to investigate the impact of fracture patterns, pinning configuration, surgeon experience and specialization on the short-term radiological prognosis in patients treated for SCHFs in the prone position.

Traditionally, the supine position is the recommended position when open reduction is required, and a planned anterior approach is mandatory for neurovascular injuries, open fractures and compartment syndromes [22]. The anterior approach can be extended medially or laterally depending on the fracture pattern or complications encountered [23]. When adequate closed reduction cannot be achieved, the superiority of one approach over another has not been established. With the posterior approach, transecting the triceps tendon from the olecranon reduces the risk of iatrogenic injury by keeping the ulnar nerve in view [24]. In our series, all patients were treated in the prone position, while 2 out of 44 patients underwent open reduction surgery for unsatisfactory reduction. On the other hand, when comparing the supine position to the prone position, a greater risk of iatrogenic ulnar nerve damage has been observed in the literature (4.5% of cases) [9,10]. The hyper-flexed elbow position during the reduction procedure in the supine position might result quicker in iatrogenic neurological damage because the ulnar nerve is hypermobile and anteriorly displaced in hyper-flexed elbows in children [9,10]. After the lateral pin insertion in the prone position, elbow flexion is not necessary [9,10].

In addition to the patient’s intraoperative position, the pin configuration choice may be based on the surgeon’s preference and experience. Literature evidence does not support a gold-standard configuration; several inconclusive biomechanical [14,15,16] and clinical [17,18,19] studies can be found in the literature [11]. 

According to a national trainee collaborative evaluation of practice, in the UK the most common configuration for Gartland type 3 fracture is the crossed-wire configuration, while lateral is the preferred fixation in 2a and 2b types [25].

In a retrospective study comparing lateral and cross-pin configurations, Pavone et al. [17] demonstrated satisfactory results with similar outcomes in terms of joint function, recovery and complications. Cross-pinning offers the most stable construct biomechanically but carries the risk of ulnar nerve injury, which is the most feared complication [17,18,19]. The medial wire insertion landmark is the little region of the medial epicondyle where the k-wire must be inserted as far anteriorly as feasible to prevent iatrogenic ulnar nerve damage [26]. The first pin was positioned in the middle, just lateral to the olecranon, and the second pin was inserted laterally in the lateral pin configuration. Although there is extremely little iatrogenic ulnar nerve damage in these situations, there have been questions raised concerning the construct’s stability [27]. The wires were spaced apart for both configurations (lateral pins and crossing pins), and particular care was taken to guarantee the pins did not cross the fracture at the same spot [17].

Whang et al. advised employing the center of the dorsal olecranon inflection (CDOI) as an anatomic reference for the proper placement of the pins in the lateral configuration in a 3D computational simulation. According to the authors, the surgeon should place the k-wires in the lower lateral quadrant composed of two ideal perpendicular lines across the CDOI in order to achieve a satisfactory stability [28]. 

In our study, patients were evenly divided into sub-groups based on the configuration; 13 children received lateral pin treatment, 20 received cross-type treatment, and 11 received lateral pin treatment in addition to medial pin treatment to increase the stability of the fracture. No differences in the pinning configurations were seen in the series for the radiological evaluation, surgical time or radiation exposure. A similar investigation comparing the configurations of a cross and two lateral and one medial (2L1M) pins was carried out by Kaya et al. [29]. The authors did not find differences between the groups in terms of clinical and radiological outcome, but in contrast to our data, they reported a higher mean surgical duration (*p* < 0.05) for the 2L1M K-wire fixation (40.61 ± 8.25 min) when compared to the crossed fixation (30.59 ± 8.72 min) and higher mean radiation duration (1.68 ± 0.55 s for the 2L1M K-wire fixation vs. 0.76 ± 0.33 s for the crossed K-wire fixation; *p* < 0.05). 

When this configuration is chosen, the goal should be to maximize pin configuration in the coronal plane. For proper stability in the distal fragment, Chong et al. proposed that the following parameters need to be optimized: ideal crossing angle (90 degrees), pin separation ratio (>0.33), distance of crossing point from fracture site and capitellar entrance [30].

However, surgery planning is mandatory for the choice of the best configuration. Wilkinson et al. modified the most widely utilized Gartland’s classification of supracondylar humerus fractures [20] and introduced the concept of posterior humeral contact. The authors separated type II into type IIA and type IIB according to the medial and lateral dislocation of the fracture [16]. A classification system that identified fracture patterns in both the coronal and sagittal planes was described by Bahk et al. in 2008 [21]. The authors identified two distinct fracture types in the sagittal plane: (a) low sagittal oblique and (b) high sagittal oblique. Low sagittal oblique fractures with a 20° angle of obliquity start anteriorly and end approximately at the same level posteriorly. High sagittal oblique fractures with an exit that is proximally and posteriorly located begin anteriorly and have an oblique angle of 20°. By combining the modified Gartland and Bahk classifications, Shah et al. [6] developed a decision algorithm to support pin configuration selection based on the fracture pattern. According to the data, the coronal fracture pattern has not been determined in the patient outcome; in fact, despite that longer surgery duration and radiological exposure were recorded in high sagittal fractures, no statistical differences were found.

In contrast to the EPOS survey respondents’ opinion [11], pediatric orthopedic surgeons did not demonstrate any superior results when compared to general orthopedics. On the other hand, fellowship-trained pediatric orthopedists may provide a faster treatment and a shorter hospital stay [31]. Several reports [32,33] have indicated that general orthopedic surgeons are familiar with the SCHF treatment. In their study, Farley et al. [34] highlighted a significantly higher rate (6% of cases) of treating the severe fractures with an open reduction for the pediatric orthopedists and suggested a higher comfort level when an open reduction is required. In a Korean consensus, Lee et al. [35] reported higher inclination in the lateral pinning technique choice for general orthopedic surgeons (*p* = 0.017) despite pediatric orthopedic surgeons and hand surgeons’ data not supporting any preference (*p* = 0.279). Furthermore, the treatment method, outcomes and complication rates were similar between orthopedic surgeons with additional pediatric training and orthopedic surgeons with different training. Similarly, the surgeon’s experience did not affect the postoperative results. In a recent study, Qian et al. [12] identified the number of 65 surgeries as the threshold for the learning curve. The cut-off decision is based on the operation time and success rate of the wire placement rather than the effectiveness of the fracture reduction or the clinical or radiological outcomes of the patients. The authors hypothesized that the inability to successfully complete the reduction or experience of multiple unsuccessful attempts at implant placement did not affect the patient’s prognosis. However, the experience accumulated from 65 operations is not the time necessary to learn the operation, but the time needed to achieve the stability of individual surgical techniques [12]. In the present series, the novice operators were supported by an experienced surgeon defined as a specialist who performed more than 15 CRPP and/or has more than 10 years of surgical experience.

### Limitations

This study has several limitations. First, the small sample size limited the ability to assess and account for all variables impacting outcomes which are multifactorial. Despite the small sample size, only two other published articles since 1951 exist with larger cohorts [36]. Future studies should include larger samples sizes to account for additional variance in patients and their impact on these radiological measurements. Furthermore, variations in radiographic quality and measurements can be found with any radiologic outcome study. This was minimized as all x-rays were conducted by two radiology technicians utilizing standardized views and techniques. Inevitably, variations in imaging and views existed and can impact our measurements and conclusions. Additionally, despite the prospective collection of data, retrospectively reviewed data allows for selection bias. On other hand, children in this study were equally distributed to each subgroup in age, sex and classification. This suggests that our study population did not suffer from selection bias.

## 5. Conclusions

Short-term radiological outcomes following the treatment of supracondylar humerus fractures treated in the prone position may not be affected by fracture patterns and pinning configuration, regardless of the surgeon’s years of experience or subspecialty.

## Figures and Tables

**Table 1:** Patient Demographics. Legend: PedOrt, pediatric orthopedic; GenOrt, general orthopedic surgeons; M, male; F, female, ExpFirst Operator, experienced first operator; NovFirst Operator, novice first operator.

Groups	Sample	Lateral Pins	Cross Pins	Others	High Sagittal	Low Sagittal	NovFirst Operator	ExpFirst Operator	PedOrt	GenOrt
Patients Number	44	13	20	11	16	28	16	28	13	31
Sex (M/F)	21/23	7/6	9/11	5/6	5/11	16/12	8/8	15/13	5/8	16/15
		*p* = 0.87			*p* = 0.09		*p* = 0.82		*p* = 0.43	
Mean Age, yrs. (stdev)	6.0 (2.5)	5.3 (3.1)	6.3 (2.0)	6.4 (2.7)	5.8 (2.6)	6.2 (2.5)	6.0 (2.5)	6.1 (2.5)	5.8 (2.3)	6.1 (2.6)
		*p* = 0.45			*p* = 0.63		*p* = 0.91		*p* = 0.66	
Side (Right/Left)	19/25	5/8	7/13	7/4	6/10	13/15	6/10	13/15	5/8	14/17
		*p* = 0.28			*p* = 0.56		*p* = 0.56		*p* = 0.68	
Mean Follow-up, yrs. (stdev)	2.7 (1.9)	3.1 (1.3)	2.5 (1.7)	2.8 (2.9)	3.2 (2.4)	2.4(1.5)	2.9 (2.6)	2.6 (1.4)	2.4 (1.7)	2.8 (2.0)
		*p* = 0.67			*p* = 0.20		*p* = 0.59		*p* = 0.51	
Unsatisfactory reduction	2	1	1	0	1	1	1	1	1	1
		*p* = 0.66			*p* = 0.68		*p* = 0.68		*p* = 0.51	

**Table 2:** Post-Operative Radiological Outcomes. Legend: PedOrt, pediatric orthopedic; GenOrt, general orthopedic surgeons, BA, Baumann’s angle; SCA, shaft-condylar angle; ExpFirst Operator, experienced first operator; NovFist Operator, novice first operator; stdev, standard deviation.

Groups	Sample	Lateral Pins	Cross Pins	Others	High Sagittal	Low Sagittal	NovFirst Operator	ExpFirst Operator	PedOrt	GenOrt
Post-op BA°(stdev)	74.8 (4.9)	73.6 (5.7)	75.9 (4.6)	74.1 (6.6)	71.6 (5.6)	75.9 (8.9)	74.3 (4.2)	75.1 (6.1)	74.9 (7.1)	74.6 (4.8)
*p*-value		*p* = 0.45			*p* = 0.10		*p* = 0.65		*p* = 0.87	
post-op SCA° (stdev)	37.7 (10.2)	40.0 (7.1)	39.4 (11.2)	32.2 (8.3)	38.3 (11.1)	37.8 (9.7)	35.0 (10.4)	39.2 (9.4)	38.1 (11.1)	36.4 (9.1)
*p*-value		*p* = 0.10			*p* = 0.89		*p* = 0.21		*p* = 0.60	

**Table 3:** Secondary Outcomes. Legend: PedOrt, pediatric orthopedic; GenOrt, general orthopedic surgeon; stdev, standard deviation; min, minutes; sec, seconds, ExpFirst Operator, experienced first operator; NovFist Operator, novice first operator.

Groups	Lateral Pins	Cross Pins	Others	High Sagittal	Low Sagittal	NovFirst Operator	ExpFirst Operator	PedOrt	GenOrt
Surgical Duration, min (stdev)	30.8 (11.5)	34.2 (37.2)	39.1 (26.3)	37.7 (43.3)	28.75 (13.0)	34.7 (24.2)	29.1 (13.6)	41.9 (44.1)	32.5 (19.6)
*p*-value	*p* = 0.78			*p* = 0.31		*p* = 0.11		*p* = 0.33	
Radiation Exposure, sec (stdev)	91.5 (74.0)	88.3 (49.5)	131.7 (81.4)	119.7 (83.1)	91.6 (57.7)	118.25 (76.0)	91.2 (61.3)	108.7 (69.9)	100.8 (69.1)
*p*-value	*p* = 0.20			*p* = 0.19		*p* = 0.07		*p* = 0.73	
Mean Number of Follow-ups	1.83 (0.94)	2.70 (1.30)	2.45 (1.12)	2.3 (1.5)	2.3 (1.09)	2.53 (1.24)	2.26 (1.16)	2.69 (1.31)	2.25 (1.23)
*p*-value	*p* = 0.12			*p* = 0.99		*p* = 0.47		*p* = 0.29	

## Data Availability

Not applicable.
